# Medical Management for the Treatment of Nontuberculous Mycobacteria Infection of the Parotid Gland: Avoiding Surgery May Be Possible

**DOI:** 10.1155/2016/4324525

**Published:** 2016-06-01

**Authors:** Emily Kay-Rivest, Sarah Bouhabel, Matthew Thomas Oughton, Michael Peter Hier

**Affiliations:** ^1^Department of Otolaryngology-Head and Neck Surgery, Jewish General Hospital, McGill University, Montreal, QC, Canada H3T 1E2; ^2^Department of Medicine, Division of Infectious Diseases, Jewish General Hospital, McGill University, Montreal, QC, Canada H3T 1E2

## Abstract

Infection with nontuberculous mycobacteria (NTM) is uncommon in the head and neck; therefore there is no clear consensus on treating these infections. Our objective was to report our experience with a unique case of NTM infection of the parotid in an immunocompetent patient, in order to determine appropriate management through our experience with this pathology. A 57-year-old man, known for numerous comorbid diseases, presented to our institution complaining of right parotid swelling and pain. A computed tomography (CT) of the neck showed a multiloculated collection in the inferior portion of the right parotid gland, compatible with abscess formation. This abscess was drained by interventional radiology (IR) but required repeat drainage twice due to lack of initial improvement. He was treated with several antibiotics as culture results initially indicated Gram-positive bacilli and then* Mycobacterium* species, with final identification by a reference laboratory as* Mycobacterium abscessus*. Imipenem was initiated with amikacin and clarithromycin. His infection clinically and radiologically resolved after 5 months of antibiotherapy. In our case, the patient improved following intravenous antibiotic therapy. Our experience demonstrates that appropriate antibiotherapy can lead to resolution of* Mycobacterium abscessus* infection in the parotid without the risks associated with surgical intervention.

## 1. Introduction

Although infection with nontuberculous mycobacteria (NTM) is uncommon in the head and neck region, isolating these organisms in the parotid gland is even less common. The parotid gland in particular, unlike other salivary glands, has lymph nodes that are within the parenchyma of the gland itself, making treatment with antibiotherapy more challenging. Parotid gland infections are usually caused by pathogens such as* Staphylococcus aureus* and anaerobic bacteria [1] and only in rare cases are secondary to a mycobacterial infection. Mycobacteria can be classified into three main groups: the bacteria that cause tuberculosis (*M. tuberculosis*), the bacteria causing leprosy (*M. leprae*), and finally all the others, collectively known as nontuberculous mycobacteria. In general, these organisms most commonly lead to pulmonary disease. It is reported that over 150 different species of NTM exist, and the most commonly discussed ones are the members of the* Mycobacterium avium* complex (MAC) [2]. A rarer species,* Mycobacterium abscessus*, is a Gram-positive, acid-fast rod that belongs to the group of rapid-growing NTM. In this report, we describe a case of a* Mycobacterium abscessus* parotid gland abscess in an immunocompetent, adult patient. We also review previous literature describing NTM infections of the parotid gland in order to attempt identification of appropriate management for this pathology. At this time, it is unclear whether surgical intervention or medical management is the preferred treatment modality.

## 2. Case Presentation

On June 5, 2014, a 57-year-old man presented to the emergency department of our institution, complaining of right parotid swelling and pain for the past two weeks. His past medical history consisted of type II diabetes mellitus, hypertension, dyslipidemia, coronary artery disease, and severe peripheral vascular disease requiring several surgical interventions and long-term anticoagulation on warfarin. He was not taking immunosuppressant medication. He reported having woken up with a large, swollen, and nonprogressive mass on the right cheek. It was described as tender and caused dysphagia (to solids more than liquids). The week prior to arrival in hospital, the patient had been treated with a seven-day course of oral penicillin following a dental procedure. It remains unclear why the patient was placed on prophylactic antibiotics following the dental procedure, as he had no history of artificial heart valves, no history of infective endocarditis, and no other congenital heart conditions. On presentation, his vital signs were normal and he was afebrile. His physical exam showed a 4 cm × 3 cm tender, nonfluctuant right neck mass just below the angle of the mandible. The rest of the physical exam was unremarkable. His white blood cell count (WBC) was slightly elevated at 12.7. HIV testing was negative. A computed tomography (CT) of the neck showed a multiloculated collection in the inferior portion of the right parotid gland, with rim and septal enhancement, compatible with an abscess formation. It showed direct involvement of the right sternocleidomastoid muscle with edematous changes and thickening. The infectious disease (ID) service was contacted and the patient was empirically started on piperacillin-tazobactam 4.5 g IV q8h, which he received until day 5 after admission. Given the CT findings, the decision was made to proceed to drainage of the abscess by interventional radiology on day 1 of admission; three milliliters (cc) of pus was drained and sent for culture, with initial results demonstrating aerobic Gram-positive bacilli. The patient did not improve following this intervention; in fact, his condition worsened and the abscess increased in size. At this time, piperacillin-tazobactam was changed to meropenem 1 g IV q8h. Vancomycin 1.25 g IV q12h was also started but stopped on day 6 after admission when screening swabs and abscess cultures were negative for methicillin-resistant* Staphylococcus aureus* (MRSA). The patient's clinical status did not improve despite the antibiotic changes and he was brought back for a second ultrasound-guided aspiration and pigtail insertion on day 5 after admission, with further 10 cc of purulent fluid being drained. Following this second intervention, the swelling improved and the patient was discharged home on meropenem IV. Blood cultures remained negative throughout. When the patient was seen in the outpatient Otolaryngology-Head and Neck Surgery (OTL-HNS) clinic on June 19, 2014 (20 days after initial presentation), a dramatic clinical improvement was noted. Although complete resolution had not yet been achieved, a major decrease in the swelling was described. There was a slight yellow-coloured ooze noted from the area. The patient was otherwise afebrile and well. The decision was made to drain the remaining fluid once again. Based on cultures staining positive for acid-fast bacilli and initial MALDI-TOF analysis consistent with* Mycobacterium* species (possibly* M. tuberculosis* or* M. bovis*), quadruple therapy for* M. tuberculosis* (isoniazid, rifampin, pyrazinamide, and ethambutol) was initiated, and the meropenem was continued pending final results. By July 7 (day 38 after initial presentation), the provincial reference laboratory identified the organism as* Mycobacterium abscessus*, and antibiotherapy was changed to imipenem 500 mg IV q6h plus amikacin 900 mg (10 mg/kg for a 90 kg patient) IV q24h. On the July 17, 2014, during a follow-up visit to the ID clinic, clarithromycin 500 mg PO BID was added to the patient's treatment regimen. This represented the standard therapy for mycobacterial infections: induction with imipenem and amikacin followed by clarithromycin treatment.

On July 27, 2014, the patient was admitted to hospital for a gastrointestinal bleed, with a supratherapeutic INR of 6.0. Infectious diseases were consulted to follow the neck mass. The ID team attributed the increase in INR to the concomitant use of broad-spectrum antibiotics along with warfarin. They suggested continuing the imipenem and amikacin, restarting clarithromycin after bleeding was controlled, and daily monitoring of his INR. Furthermore, a work-up was done for immunodeficiencies but all tests came back as normal. All serologies were also found to be negative. Amikacin was subsequently stopped after one month due to ototoxicity (mild loss of ultrahigh frequency range hearing) detected on serial audiometry. However, imipenem and clarithromycin were continued for an additional four months. Serial CT imaging of his neck in November and December showed complete resolution of the fluid components of the abscess.

## 3. Discussion

As noted in the few other existing case reports, nontuberculous mycobacterial infection of the parotid gland is quite rare, especially in adults who are immunocompetent. Most of the literature describing this pathology is within the field of pediatrics or among adults with HIV infection. Very few case reports describe immunocompetent adults with NTM infection of the parotid.

In our case, despite the fact that our patient was not immunocompromised, the diagnosis of diabetes mellitus is significant, as it is a well-known risk factor for developing parotitis [3]. It is thought that diabetes may lead to “changes in the composition of saliva, duct obstruction, and reduced immunity,” making the patient more susceptible to infections of the parotid gland [4].

It remains difficult to assess what may have led to this unusual infection in this immunocompetent patient. Some reports suggest that the most likely cause of infection is by direct ingestion of the pathogen [5]. NTM are found throughout the environment, in water, food, and soil. As ingestion is a possible mechanism of infection, this may explain why children are more at risk of contracting these types of infections. Interestingly, in April 2016 the Centers for Disease Control and Prevention published a review of* M. abscessus* infections among patients of a pediatric dentistry practice in Georgia, USA. They describe a cluster of 9 cases of confirmed or suspected infections related to common source exposure: water rinse in the dentist's office [6]. Nonetheless, despite this isolated event, no other reported contemporaneous cases are present in the literature. We do not believe that the dental procedure that he underwent prior to developing this infection was the cause. It is also unclear why MALDI-TOF analysis identified this organism as possible* M. tuberculosis* or* M. bovis*, as these slow-growing mycobacteria are significantly different from the rapid-growing* M. abscessus*; an error in sample processing or preparation may have occurred.

Many different treatment modalities for NTM infection of the parotid gland lymph nodes are described in the few existing reports. Shah and Haddad Jr. report that surgical excision of the parotid gland is considered more effective than antibiotic treatment [7]. In their experience, they describe three patients with NTM parotid adenitis (all children between 15 and 30 months of age). In two of their patients, disease improvement was not seen with prolonged antibiotherapy and only resolved after surgical intervention. In the third patient, surgical excision was planned from the start, with a preoperative course of clarithromycin and good postoperative resolution. In another case series performed by Lindeboom et al. (done in children and encompassing all cervicofacial NTM infections), surgical removal of tissues infected with NTM was successful in 96% of patients, compared to a 66% rate of complete regression when the infection was treated with antibiotics alone [8]. Furthermore, only one report published showed successful results with a combination of abscess drainage and antibiotic treatment [9]. In our case, initial drainage by interventional radiology along with antibiotic treatment resulted in slow resolution. The patient required repeated drainage in interventional radiology and resolved with long-term intravenous antibiotic therapy. The patient did achieve complete resolution, despite the delay in pathogen identification and proper treatment. Although several case reports describe better outcomes with surgical intervention preceded by antibiotherapy, complete surgical excision of a parotid gland is not without risks, particularly in an abscessed gland. There always exists the chance of injury to the facial nerve, which can leave the patient with permanent hemifacial paralysis. There is also the risk of Frey syndrome, numbness along the distribution of the greater auricular nerve, bleeding and hematoma, and seroma formation. On the other hand, certain antibiotics given in the long term can be associated with side-effects such as ototoxicity (amikacin). Overall, it is necessary to weigh the risks of surgery compared to its benefits, but combined antimicrobial therapy appears to be a reasonable alternative to surgery.

## 4. Conclusion

As mentioned above, NTM infection of the parotid gland is a rare entity. To our knowledge, a total of five case reports exist describing this type of infection in adults, with only two in an immunocompetent individual ([4, 10–13], summarized in Table 1). Given the rarity of the disease, there is no established “gold standard” at this time for treatment. The literature proposes that a combined surgical and medical approach with close follow-up may be the preferred method for management of NTM infections of the parotid gland. However, our experience demonstrates that using appropriate antibiotherapy can also lead to resolution without the risks associated with surgery.

## Figures and Tables

**Table 1:** Summary of case reports of NTM parotid infection in *adults*.

Study	Number of cases	Bacteria	Comorbid disease	Treatment modality
Benharrats et al. 1998 [11]	1 case	*M. xenopi*	HIV positive	Antibiotics alone
Lawn et al. 2005 [10]	1 case	*M. scrofulaceum*	HIV positive	Antibiotics alone
Gittinger et al. 2008 [4]	1 case	*M. avium*	Rheumatoid arthritis, DMII secondary to steroids	Surgical excision and then antibiotics
Padovani et al. 2007 [13]	1 case	NTM	None cited	Surgical excision
Yamanaka et al. 2013 [12]	1 case	*M. fortuitum or M. chelonae (suspected)*	None	Antibiotics alone
